# Molteno3 Implantation as Primary Glaucoma Surgery

**DOI:** 10.1155/2014/167564

**Published:** 2014-03-11

**Authors:** Juha O. Välimäki, Ari-Pekka A. Ylilehto

**Affiliations:** Department of Ophthalmology, Päijät-Häme Central Hospital, Keskussairaalankatu 7, FIN-15850 Lahti, Finland

## Abstract

*Purpose*. To determine the outcome of Molteno3 implantation as primary glaucoma surgery and to analyze the factors influencing the surgical outcome. *Methods*. This is a retrospective clinical study of 106 consecutive eyes (97 patients) with no previous glaucoma surgery. Surgical failure was defined as an IOP > 21 mmHg or less than a 20% reduction below baseline, or IOP ≤ 5 mmHg, on two consecutive visits after 3 months follow-up, or reoperation for glaucoma or loss of light perception. *Results*. At the end of the follow-up (mean, 35 months; range, 12–71 months), the mean postoperative IOP (14.2 ± 4.4 mmHg) was statistically significantly lower than the preoperative IOP (35.2 ± 9.7 mmHg) (*P* < 0.001). Life-table success rates were 97%, 94%, and 91% after follow-up of 12, 24, and 36 months, respectively. Success rate for an IOP ≤ 18 mmHg was 77% at the last visit. Success was not influenced by previous cataract surgery, sex, age, laser trabeculoplasty (LTP), preoperative IOP, or number of antiglaucoma medications. Forty-seven eyes had 66 postoperative complications. *Conclusions*. The primary Molteno3 implant provided significant IOP lowering with minimal and manageable complications in uncontrolled glaucoma. Neither previous cataract surgery nor LTP had any detrimental effect on surgical success.

## 1. Introduction

Glaucoma drainage implant (GDI) is typically reserved for patients in whom filtration surgery has been unsuccessful. A great deal of clinical data has been published on GDI surgery in refractory glaucoma when prior surgical therapy has failed [1–4]. A Tube versus Trabeculectomy (TVT) study has shown that in patients who do not yet have refractory glaucoma GDI surgery is as efficacious as trabeculectomy [5]. However, in that TVT study, some eyes in the tube group had undergone failed trabeculectomy before the GDI procedure.

Although GDI surgery has become more common [6], little is known about its usefulness as primary glaucoma surgery beyond refractory cases. Wilson et al. showed that GDI surgery could be used as the primary procedure in cases of advanced primary open angle glaucoma (POAG) and advanced chronic closed angle glaucoma (CCAG) [7]. Vuori reported good results in a case series of uveitic glaucoma patients receiving GDI as primary surgery [8].

The purpose of this study was to determine the outcome of Molteno3 implantation as primary glaucoma surgery and to evaluate the factors influencing the surgical outcome, especially the effect of previous cataract surgery.

## 2. Materials and Methods

Data on 106 eyes of 97 consecutive patients (53% male) were retrospectively reviewed, including all patients treated with Molteno3 (Molteno Ophthalmic Limited, Dunedin, New Zealand) implantation as primary surgical treatment for glaucoma between August 17 2007 and April 19 2012 at Päijät-Häme Central Hospital, Lahti, Finland. No previous glaucoma surgery, including cyclophotocoagulation, had been performed in any of the eyes included in the study, but prior laser trabeculoplasty (LTP) was accepted. Eyes that had previously been subjected to cataract surgery were included. Four eyes had previous pars plana vitrectomy. Every eye included had not intraocular surgery within 3 months prior Molteno3 implantation.

GDI surgery was indicated when the visual function of a patient on maximum tolerated antiglaucoma medication and with LTP, if indicated, was failing or likely to fail at current level of intraocular pressure (IOP), and in case of open angle glaucoma cataract surgery had been performed or the patient's other eye had previously undergone unsuccessful trabeculectomy; or, in the case of chronic angle-closure glaucoma, both prior YAG laser iridotomy and cataract surgery had been performed.

The study population was divided into two different subgroup settings for analysis. In setting A, group 1 consisted of all eyes with POAG and pseudoexfoliative glaucoma (PEXG) and group 2 eyes with uveitic glaucoma. In setting B, group 3 consisted of eyes without previous cataract surgery and group 4 eyes with previous cataract surgery.

### 2.1. Surgical Technique

All patients underwent single-stage, single-plate 175 mm^2^ Molteno3 implant surgery. The procedure for each patient was performed by one of the authors (Välimäki) using the same technique as described in our earlier study [9]. In brief, all implants were inserted in one stage into the superior temporal quadrant. The anterior edge of the plate was fixed to the sclera 8 to 10 mm posterior to the limbus between the rectus muscles. One 6–0 polyglactin (Vicryl Rapide, Ethicon) ligature around the tube was used in all eyes to close the tube for the first approximately two to four weeks. Balanced salt solution was irrigated via the tube to confirm tube ligation status after ligature placement. The tube was inserted into the anterior chamber through a 23-gauge puncture under the scleral flap. The extraocular part of the tube was led through the scleral tunnel.

No scleral patch graft—either 5-fluorouracil (5-FU) or mitomycin C (MMC)—was used. The conjunctival incisions were closed with an interrupted 8–0 silk suture. The postoperative medical regimen included a combination of topical antibiotics and corticosteroids five times daily for the first four to five weeks.

### 2.2. Follow-Up

All patients were followed up for at least 12 months. Check-ups were made on the first postoperative day, two weeks, one month, three months, and six months after surgery and subsequently according to patient status. Status at the last follow-up visit was used in the study. At every check-up, Snellen visual acuity (VA) and IOP were measured and slit-lamp biomicroscopy and ophthalmoscopy were performed. Antiglaucoma medication was adjusted during the visit if necessary. Any complications observed were managed according to the situation. All additional surgery during the follow-up period was recorded.

### 2.3. Outcome Measures

Outcome measures included IOP, VA, number of antiglaucoma medications, any surgical complications, and failure. Surgical failure was defined as in the TVT study: IOP > 21 mmHg or less than 20% reduction below baseline or IOP ≤ 5 mmHg, on two consecutive visits after 3 months' follow-up, reoperation for glaucoma, or loss of light perception [5]. We also considered failure rate with more stringent IOP limits; IOP > 18 mmHg and IOP > 14 mmHg. Eyes that had not failed according to the above criteria and that did not require supplemental medical therapy were considered complete successes. Eyes that had not failed but which required supplemental antiglaucoma medication were defined as qualified successes. Total success refers to both complete and qualified success. The influence of preoperative factors (sex, lens status, age, preoperative IOP, preoperative number of antiglaucoma medications, and previous LTP) on postoperative IOP and GDI surgery success rate was evaluated. Humphrey visual field central 24–2 threshold test (Humphrey Field Analyzer HFA 740i, Carl Zeiss Meditec Inc., Dublin, CA, USA) indices of mean deviation and pattern standard deviation were collected from the examinations performed preoperatively and at the end of the follow-up.

### 2.4. Statistical Analysis

Comparisons were made using a* t*-test and analysis of variance with Scheffe's test for continuous data with normal distribution, Wilcoxon's signed-rank test and independent samples Mann-Whitney *U* test for continuous data without normal distribution, and Pearson's *χ* ^2^ for categorical data. Correlations were analysed using Pearson's correlation test. Surgical success was represented by means of Kaplan-Meier survival curves and groups were compared using a log-rank test. Statistical significance was implied by a *P* value less than or equal to 0.05 except for Pearson correlation *P*  values of less than or equal to 0.01.

## 3. Results

In this study 106 eyes of 97 patients were followed up for a mean of 35.0 ± 17.0 months (range 12–71 months).  Table 1 shows the preoperative demographic data of the study group. In setting A group 1 and group 2 differed from each other (Table 1). In setting B group 3 and group 4 were comparable (Table 1). Three eyes needed reoperation for glaucoma and two patients (two eyes) died during the first year of follow-up and were excluded from postoperative comparisons.

### 3.1. Intraocular Pressure

The mean (±SD) preoperative IOP was 35.2 ± 9.7 mmHg (range 18–56) and the mean postoperative IOP at the last follow-up visit was 14.2 ± 4.4 mmHg (range 0–22), the pressure drop being 20.9 ± 10.8 mmHg (59%) (*P* < 0.001; 95% CI 18.7–23.0, paired samples* t*-test). The mean postoperative IOP in the subgroups was 15.4 ± 5.0 in group 1, 12.9 ± 4.4 in group 2, 14.6 ± 4.4 in group 3, and 13.9 ± 4.5 in group 4. The mean number of antiglaucoma medications decreased from 4.0 ± 1.1 preoperatively to 1.3 ± 1.3 postoperatively at the last follow-up visit; a reduction was 2.7 ± 1.7 (*P* < 0.001; 95% CI 2.4–2.0, paired samples* t*-test).

There was no correlation between preoperative and postoperative IOP (*P* = 0.406, Pearson), number of preoperative antiglaucoma medications and postoperative IOP (*P* = 0.707), or age and postoperative IOP (*P* = 0.156). Postoperative IOP was not influenced by previous LTP (*P* = 0.362, analysis of variances), sex (*P* = 0.232), or lens status (*P* = 0.927).

### 3.2. Success Rate of Surgery

**Figure 1:**
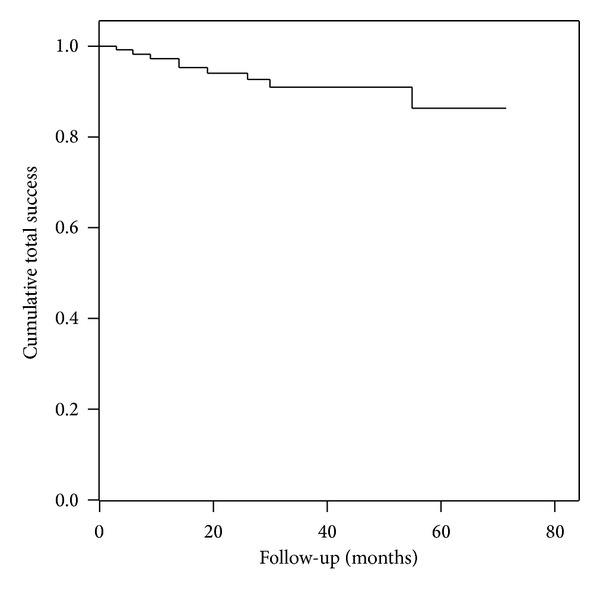
Life-table curve of cumulative total success rates for 106 eyes treated with Molteno3 implantation as primary glaucoma surgery.

Kaplan-Meier life-table analysis showed cumulative success rates of 97%, 94%, and 91% after follow-up of 12, 24, and 36 months, respectively (Figure 1). On the last follow-up visit, 38 eyes (36%) were considered complete successes, 57 (54%) qualified successes, and 11 eyes (10%) were considered as failures. Using the stringent 18 mmHg IOP limit the numbers were 32 (30%), 50 (47%), and 24 (23%) and with the 14 mmHg limit 19 (18%), 29 (27%), and 58 (55%), respectively.

**Figure 2:**
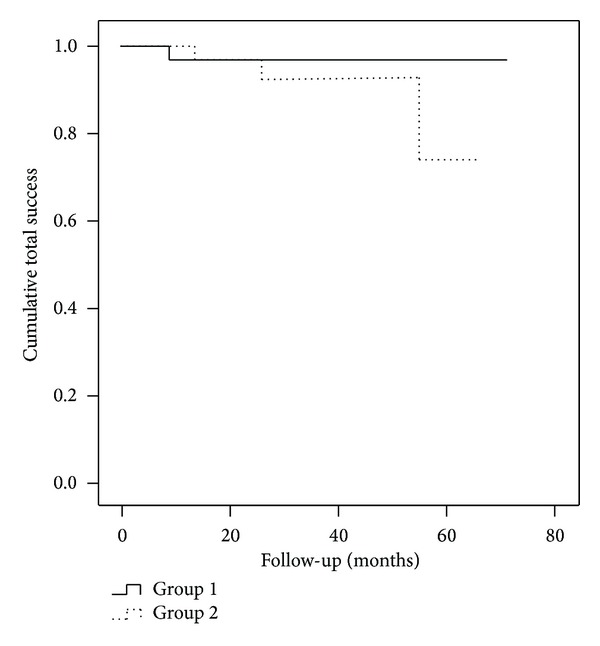
Life-table curves of cumulative total success rates in group 1 (primary open angle glaucoma and pseudoexfoliative glaucoma patients, *n* = 38) and in group 2 (uveitic glaucoma patients, *n* = 41). The difference calculated for the entire follow-up period was not statistically significant (log-rank *P* = 0.223).

**Figure 3:**
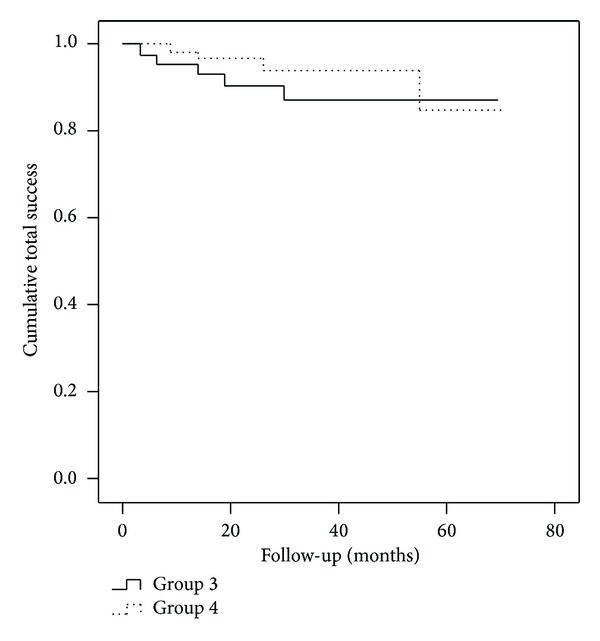
Life-table curves of cumulative success rates in group 3 (no previous cataract surgery, *n* = 45) and in group 4 (previous cataract surgery, *n* = 61). The difference calculated for the entire follow-up period was not statistically significant (log-rank *P* = 0.451).

The cumulative total success rate did not differ statistically significantly between group 1 and group 2 (log-rank *P* = 0.223) (Figure 2) and group 3 and 4 (log-rank *P* = 0.451) (Figure 3). The complete success rates were 26%, 51%, 36%, and 36% in groups 1, 2, 3, and 4, respectively. Reasons for failures were listed in Table 2. Success rate was not influenced by sex (*P* = 0.851, Pearson's *χ* ^2^ test), lens status (*P* = 0.753), previous LTP (*P* = 0.327), age (*P* = 0.923, analysis of variances), preoperative IOP (*P* = 0.562), or number of preoperative antiglaucoma medications (*P* = 0.851).

### 3.3. Visual Acuity and Perimetry

The log MAR VA was preoperatively 0.325 and deteriorated postoperatively to 0.474; however, reduction was not statistically significant (*P* = 0.169, related samples Wilcoxon's signed-rank test). VA remained within 1 line in 59 eyes (60%), improved in 16 eyes (16%), and deteriorated in 23 eyes (24%). Postoperatively log MAR VA was 0.464 in eyes without remarkable cataract formation and 0.567 in eyes with remarkable cataract formation considered as complication (*P* = 0.658, independent samples Mann-Whitney *U*). Preoperative VA was not measured in four eyes because of young age or mental disability. One of these four eyes suffered from retinal detachment and redetached gaining grade V level, postoperative VA being recorded as no light perception. Out of three hypotonic eyes one resulted in phthisis bulbi and loss of light perception, one lost five Snellen lines, and one gained one line.

Central 24–2 threshold test Humphrey visual field examination was performed preoperatively in 50 (47%) of 106 eyes and postoperatively in 39 (37%) eyes. An average preoperative mean deviation was −11.3 ± 7.4 dB and −12.1 ± 7.2 dB at the end of follow-up (*P* = 0.017, paired samples* t*-test). An average preoperative pattern standard deviation was 7.1 ± 3.5 dB and 7.8 ± 3.7 dB at the end of follow-up (*P* = 0.018, paired samples* t*-test). Visual field was analyzed with Goldmann perimeter due to poor vision preoperatively in 6 eyes and postoperatively in 8 eyes. There were five eyes with no preoperative or postoperative visual field examination available due to patients' young age or disability. Visual field examination was performed outside our hospital and was not available preoperatively in 45 eyes and postoperatively in 54 eyes.

### 3.4. Complications

No intraoperative complications were seen in the present study. There were postoperative complications in 47 eyes (44%). Early hypotony, that is, IOP ≤ 5 mmHg on any follow-up visit during the first postoperative month, was found in 20 eyes and was the most common complication. Two eyes with early hypotony were failing on the last follow-up visit, one due to hypotony and one due to reoperation for glaucoma related to persistent hypertony. All postoperative complications are listed in Table 3. No case of choroidal effusion needed an operation in the study population. During the study period, three eyes were treated by means of cataract removal. Managing the postoperative complications required 16 operations in 15 eyes which are listed in Table 4. Anti-VEGF injection was used for macular edema, in one of which the aetiology was diabetes. Vitrectomy was performed in two eyes due to malignant glaucoma and in one eye due to retinal detachment. At the last follow-up visit, one eye with persistent bullous keratopathy was referred for penetrating keratoplasty and one eye with tube erosion for repositioning of the tube. In addition, in one eye tube exposure was not possible to be repaired because of aggressive scleritis.

## 4. Discussion

In the present study, the success rate of Molteno3 implantation as primary glaucoma surgery was good and the number of severe complications low at intermediate follow-up. Good IOP control with a decreased need for medication was achieved with most patients and visual acuity was preserved. Our study showed that neither previous cataract surgery nor laser trabeculoplasty had any detrimental effect on the surgical success rate or postoperative IOP in primary GDI surgery, nor did the patient's sex, age, or preoperative IOP. Weaknesses of the study are that it is retrospective, it is not randomised or controlled, and follow-up period is only intermediate. In addition, remarkable proportion of visual field examination data was not available.

In an earlier report of 59 patients with advanced POAG or CACG who had received an Ahmed implant as primary surgery the success rate was 87.9% after a follow-up period of 11 to 13 months and 69.8% after a follow-up period of 41 to 52 months [7]. Generally speaking, the success rate of GDI surgery has been reported to decrease by 10% per year [10]. When GDI studies are carried out on eyes prior to the refractory phase of glaucoma, better success rates might be reported than before. In the TVT study the success rate in the tube group was 96.1% after one year and 70.2% after five years [5]. However, we cannot generalize the TVT study results to cover patients with GDI as primary surgery, because some eyes in that study had previously undergone unsuccessful trabeculectomy.

In our previous study, the success rate of surgery was 71% after 12 months, that is, much lower than in the present study (97%) [9]. In that earlier study, 36% of patients had previously undergone unsuccessful glaucoma surgery or diode laser cyclophotocoagulation. In both studies, almost half of the patients had undergone cataract surgery. Our present study included more uveitic glaucoma patients than in the previous study (39% versus 27%, resp.) and fewer neovascular glaucoma patients (8% versus 10%, resp.), but the differences cannot fully explain the difference in success rate. In the study of Thompson and coworkers, 17% of 87 eyes with Molteno3 implant had previously undergone trabeculectomy [11]. They reported successful IOP control in 79% at 3 years, which is, less than in our study with near same IOP criteria. Thus, this might indicate that previous glaucoma surgery adversely affects the outcome of GDI surgery.

In the case of uveitic glaucoma patients who had undergone surgery with primary Molteno implantation alone, the success rates are reported to be even better: 97% after one year and 85% after four years [8]. Our total success rate of 91% after a follow-up of 36 months in all study populations was within the same range as reported by Vuori [8]. In the present study, no differences in success rates were observed between uveitic glaucoma patients and the group of POAG and PEXG patients. This might indicate that primary Molteno3 implantation is as suitable for the uveitic glaucoma patients as for the POAG and PEXG patients. However, some degree of bias is possible because these two study groups were not comparable.

The change in VA in our study was comparable to that reported by Broadway and coworkers [12]. They also described a significant risk of failure in GDI surgery related to pseudophakia [12]. In our study we found no difference in success rates according to whether cataract surgery had previously been performed or not. Neither did lens status, including aphakia, affect postoperative IOP nor success of surgery. On the other hand, aphakic patients were a minor entity in our study (3% versus 25% in Broadway's study).

In the TVT study, deterioration in VA was mostly related to glaucoma [13]. An average preoperative mean deviation on Humphrey visual field testing was slightly better in our patients than in the TVT study tube group at baseline (−11.3 versus −16.0, resp.) [5]. This difference might indicate that fewer patients had advanced stage glaucoma in our study than in the TVT study tube group. However, we had much more missing Humphrey visual field examinations than the TVT study (42% versus 16%, resp.). A slight but statistically significant reduction in visual field mean deviation and pattern standard deviation took place. The relatively short-term nature of our study had the confounding influence to the defining of the visual field progression.

In this study, a total of 66 postoperative complications were noted in 47 eyes (44%). The incidence of complications was comparable to that reported by the TVT study [13]. The most common complication was early hypotony (19%). This supported our earlier result with Molteno3 that this dual-chamber implant does not seem to reduce the risk of postoperative hypotony [9]. Early hypotony is usually related to choroidal effusion and a flat anterior chamber, which were common complications in our study as in previous studies [7, 12–14]. In our study these complications were mainly transient and self-limiting; only in one eye anterior chamber reformation was needed.

During our study period, two eyes developed bullous keratopathy. Central corneal endothelial cell density (CCED) has been found to decrease with time after GDI surgery [15]. The same study also showed that the number of previous operations is related to a decrease in CCED. Our study period was of only medium duration and the number of earlier operations was small. This could explain why bullous keratopathy was not as common in this study as in the studies mentioned above [7, 12–14]. No endophthalmitis or blebitis was found in any of our subjects during the study period. One patient complained of transient diplopia,although no diplopia tests were performed during the follow-up visits.

In conclusion, the primary Molteno3 implant provided significant IOP lowering with minimal and manageable complications in uncontrolled glaucoma eyes with no previous glaucoma surgery. Neither previous cataract surgery nor laser trabeculoplasty had any detrimental effect on surgical success. No differences in success rates were observed between uveitic glaucoma patients and the group of POAG and PEXG patients.

## Figures and Tables

**Table 1:** Demographic data of study population and differences in demographic data between group 1 (primary open angle glaucoma and pseudoexfoliative glaucoma, *n* = 38) and group 2 (uveitic glaucoma, *n* = 41) and group 3 (no previous cataract surgery, *n* = 45) and group 4 (previous cataract surgery, *n* = 61).

Variable	All eyes	Group 1	Group 2	*P*	Group 3	Group 4	*P*
Age (y)	51.3 ± 22.6	66.8 ± 13.5	36.4 ± 18.9	<0.001	47.4 ± 22.6	54.1 ± 22.4	0.134
IOP (mmHg)	35.2 ± 9.7	31.2 ± 8.4	39.1 ± 8.8	<0.001	34.4 ± 9.9	35.8 ± 9.6	0.494
*No. meds	4.0 ± 1.1	3.8 ± 1.1	4.3 ± 0.8	0.014	3.8 ± 1.2	4.1 ± 1.0	0.137
LTP (%)	34	79	2	<0.001	38	31	0.476
^†^Lens (%) P : PP : AP	41 : 56 : 3	47 : 53 : 0	34 : 61 : 5	0.229	—	—	—
Sex M : F (%)	53 : 47	47 : 53	51 : 49	0.732	56 : 44	51 : 49	0.629

IOP: intraocular pressure.

*Number of antiglaucoma medications.

LTP: laser trabeculoplasty.

^†^Lens: P: phakic; PP: pseudophakic; and AP: aphakic.

**Table 2:** Primary reason for surgical failure.

Reason for failure	*n* (%)
Hypotony	4 (4)
Reoperation of glaucoma	3 (3)
Hypertony	2 (2)
Loss of light perception	2 (2)

**Table 3:** All postoperative complications in 106 eyes of 97 patients.

Complication	*n* (%)
Early hypotony*	20 (19)
Cataract	9 (9)
Choroidal effusion	8 (8)
Tube erosion through conjunctiva	4 (4)
Flat anterior chamber	4 (4)
Tube contact with iris/endothelium	4 (4)
Encapsulated bleb	3 (3)
Bullous keratopathy	2 (2)
Macular oedema	2 (2)
Malignant glaucoma	2 (2)
Transient diplopia	1 (1)
Retinal detachment	1 (1)
Phthisis bulbi	1 (1)
Central retinal vein occlusion	1 (1)
Wound leak	1 (1)
Exacerbation of chronic uveitis	1 (1)
Silicone oil in anterior chamber	1 (1)
Corneal abrasion	1 (1)
Total number of complications	66
^†^Eyes with complications	47 (44)
Mean number of complications/eyes	1.4

*IOP ≤ 5 mmHg on any follow-up visit during the first postoperative month.

^†^More than one complication may be present in one eye.

**Table 4:** Operations performed to treat complications.

Operation	*N*
Cataract removal	3
Vitrectomy	3
Anti-VEGF injection intravitreally	2
Repair of tube exposure	2
Anterior chamber reformation	1
Penetrating keratoplasty	1
Steroid implant injection	1
Alcohol injection	1
Removal of silicone oil from anterior chamber	1
Surgical tube ligature release	1
Total number of operations	16
Eyes operated on*	15 (14%)

*More than one operation may be present in one eye.

## References

[1] Minckler DS, Heuer DK, Hasty B, Baerveldt G, Cutting RC, Barlow WE (1988). Clinical experience with the single-plate Molteno implant in complicated glaucomas. *Ophthalmology*.

[2] Freedman J, Rubin B (1991). Molteno implants as a treatment for refractory glaucoma in black patients. *Archives of Ophthalmology*.

[3] Lloyd MA, Sedlak T, Heuer DK (1992). Clinical experience with the single-plate Molteno implant in complicated glaucomas: update of a pilot study. *Ophthalmology*.

[4] Välimäki J, Tuulonen A, Airaksinen PJ (1998). Outcome of Molteno implantation surgery in refractory glaucoma and the effect of total and partial tube ligation on the success rate. *Acta Ophthalmologica Scandinavica*.

[5] Gedde SJ, Schiffman JC, Feuer WJ, Herndon LW, Brandt JD, Budenz DL (2012). Treatment outcomes in the tube versus trabeculectomy (TVT) study after five years of follow-up. *American Journal of Ophthalmology*.

[6] Ramulu PY, Corcoran KJ, Corcoran SL, Robin AL (2007). Utilization of various glaucoma surgeries and procedures in medicare beneficiaries from 1995 to 2004. *Ophthalmology*.

[7] Wilson MR, Mendis U, Paliwal A, Haynatzka V (2003). Long-term follow-up of primary glaucoma surgery with ahmed glaucoma valve implant versus trabeculectomy. *American Journal of Ophthalmology*.

[8] Vuori M-L (2010). Molteno aqueous shunt as a primary surgical intervention for uveitic glaucoma: long-term results. *Acta Ophthalmologica*.

[9] Välimäki J (2012). Surgical management of glaucoma with molteno3 implant. *Journal of Glaucoma*.

[10] Minckler DS, Francis BA, Hodapp EA (2008). Aqueous shunts in glaucoma. A report by the American Academy of Ophthalmology. *Ophthalmology*.

[11] Thompson AM, Molteno AC, Bevin TH, Herbison P (2013). Otago glaucoma surgery outcome study: comparative results for the 175-mm2 Molteno3 and double-plate Molteno implants. *JAMA Ophthalmology*.

[12] Broadway DC, Iester M, Schulzer M, Douglas GR (2001). Survival analysis for success of Molteno tube implants. *British Journal of Ophthalmology*.

[13] Gedde SJ, Herndon LW, Brandt JD, Budenz DL, Feuer WJ, Schiffman JC (2012). Postoperative complications in the tube versus trabeculectomy (TVT) study during five years of follow-up. *American Journal of Ophthalmology*.

[14] Budenz DL, Barton K, Feuer WJ (2011). Treatment outcomes in the Ahmed baerveldt comparison study after 1 year of follow-up. *Ophthalmology*.

[15] Hau S, Scott A, Bunce C, Barton K (2011). Corneal endothelial morphology in eyes implanted with anterior chamber aqueous shunts. *Cornea*.

